# Presence of trypanosomatids, with emphasis on *Leishmania*, in Rodentia and Didelphimorphia mammals of a rural settlement in the central Amazon region

**DOI:** 10.1590/0074-02760200427

**Published:** 2021-07-09

**Authors:** Genevere Reis Achilles, Rafael Pinto Kautzmann, Haile Dean Figueiredo Chagas, Jordam William Pereira-Silva, Jéssica Feijó Almeida, Fernanda Rodrigues Fonseca, Maria Nazareth Ferreira da Silva, Felipe Arley Costa Pessoa, Alessandra Ferreira Dales Nava, Claudia María Ríos-Velásquez

**Affiliations:** 1 Fundação Oswaldo Cruz-Fiocruz, Instituto Leônidas e Maria Deane, Programa de Pós-Graduação em Condições de Vida e Situações de Saúde na Amazônia, Manaus, AM, Brasil; 2 Universidade Federal do Amazonas, Programa de Pós-Graduação em Zoologia, Manaus, AM, Brasil; 3 Fundação Oswaldo Cruz-Fiocruz, Instituto Leônidas e Maria Deane, Laboratório de Ecologia de Doenças Transmissíveis na Amazônia, Manaus, AM, Brasil; 4 Instituto Nacional de Pesquisa da Amazônia, Laboratório de Genética Animal, Manaus, AM, Brasil

**Keywords:** Leishmania, Trypanosoma, reservoirs, rodent, marsupials, anthropisation

## Abstract

**BACKGROUND:**

Trypanosomatids are widespread and cause diseases - such as trypanosomiasis, sleeping sickness, Chagas disease, and cutaneous and visceral leishmaniasis - in animals and humans. These diseases occur in both rural and urban regions due to unplanned growth and deforestation. Thus, wild and synanthropic reservoir hosts living in residential areas are risk factors.

**OBJECTIVE:**

We aimed to evaluate the diversity of small mammals (rodents and marsupials), and the occurrence of trypanosomatids, especially *Leishmania*, in the rural settlement of Presidente Figueiredo, Amazonas.

**METHODS:**

Animals were collected using Sherman, Tomahawk, and Pitfall traps along 16 trails in four landscapes: continuous forest, forest with planting, planting, and peridomiciliar. *Leishmania* sp. was detected in liver samples by polymerase chain reaction targeting kDNA.

**FINDINGS:**

Diversity was higher in forests with planting and lower around residences. In total, 135 mammals (81 rodents and 54 marsupials covering 14 genera) were captured. Rodents presented infection rates (IR) of 74% and marsupials of 48%. Rodents in domicile landscapes presented a higher IR (92.9%), while marsupials showed a higher IR in forests (53.3%).

**MAIN CONCLUSIONS:**

The results suggest high prevalence of trypanosomatids across 12 mammalian genera possibly involved as reservoir hosts in the enzootic transmission of leishmaniasis in the Amazon’s rural, peridomiciliar landscape.

Chagas disease (CD) and American Tegumentary Leishmaniasis (ATL) are neglected zoonotic diseases that are endemic to Brazil, particularly in the northern region of the country where their incidences are higher due to unplanned urbanisation of land by human occupation. Between 2000 and 2013, Brazil confirmed 1,570 CD cases, 91.1% of which were reported in the north region;^1^ in 2015, there were 8,939 cases in the north, corresponding to 46% of the total cases reported across the country.^2^

These diseases affect the rural and peripheral populations of cities, generally in socioeconomically deprived places with low housing and sanitary support, and difficult of access to healthcare facilities.^3^ The diseases present wild and peridomiciliar;^4^ for instance, occasional breakouts in suburban areas of Manaus were reported due to unplanned occupation in Comunidade São João (17 cases of ATL) and in the communities of Ramal de Iporar (Rio Preto da Eva), Tarumã Mirim, and the east zone of Manaus (147 cases of ATL).^4^ ^,^ ^5^

In most of these breakouts, vector insects and species of *Leishmania*, which cause leishmaniasis, have been well studied; however, little is known about the natural reservoirs, which are usually small mammals of the orders Rodentia and Didelphimorphia living in wild and synanthropic environments.^6^ ^,^ ^7^ For instance, the first case of *Leishmania amazonensis* in *Rattus rattus* was described in the State of Paraná, indicating the possible urbanisation of ATL in that area.^7^ Further, infected common opossum (*Didelphis marsupialis*) individuals were found in the peripheral regions of Manaus,^8^ ^,^ ^9^ which was undergoing urbanisation, with infection rates (IR) over 20% for *L. guyanensis*. *D. marsupialis* have also been reported with *Trypanosoma cruzi* and *Leishmania* sp. in Salvador, Bahia.^10^

Although some studies have indicated natural *Leishmania* infections in certain species of rodents and marsupials, few studies have been conducted using an ecological approach, which is fundamental in to understand the role of each species in the parasite’s maintenance and life cycle. Elucidation in this regard can lead to suitable applications of the information such as to identify and implement new strategies in the control of diseases. The objective of this study was to evaluate the diversity of small mammals, rodents, and marsupials, and the occurrence of trypanosomatids - with emphasis on *Leishmania* - across four distinct landscapes in the rural settlement of Presidente Figueiredo in the central Amazon region.

## MATERIALS AND METHODS

**Fig. 1:**
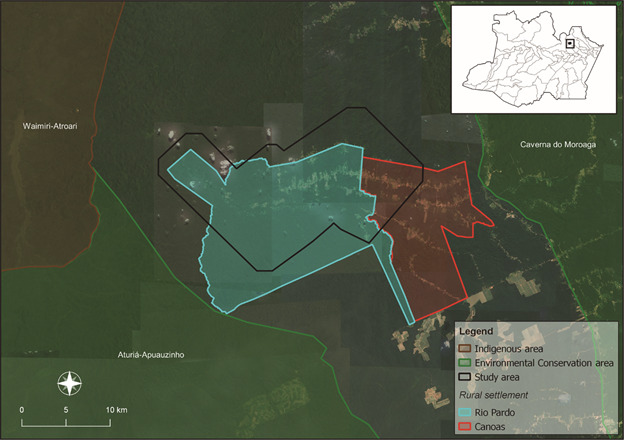
outlined map of rural settlement of Rio Pardo, municipality of Presidente Figueiredo - Amazonas State. The area in blue is Rio Pardo settlement area and in red is Canoas settlement. The settlements area are included households and settlement forest area. The area sketchy in black is the study site.

*Field work* - This study was conducted in the rural settlement of Rio Pardo (RSRP), municipality of Presidente Figueiredo, Amazonas (AM), located 160 km from Manaus city (Fig. 1). The range of the settlement is 27.980 ha, and it is occupied by approximately 600 people, as reported in the last census (2015) conducted by the Instituto Leônidas e Maria Deane (ILMD).

The RSRP was originally created by the National Institute for Colonisation and Agrarian Reform (INCRA) in 1996^11^ in an area of dense tropical forest and solid ground. With a tropical-humid weather profile, the region has an average annual temperature of 27ºC and two distinct climatic periods: a rainy season (November to May) and a dry season (June to October).

*Capture and identification of animals* - A total of four expeditions were conducted (Table I). Small ground mammals were captured in four landscapes of distinct anthropisation levels: peridomiciliar, planting, forest with planting, and continuous forest. Continuous forest refers to a landscape with less or no anthropic effects, only touched by plant extractivism, i.e., fishing and hunting; forest next to planting refers to forested areas that are regenerating due to the anthropic interference of the inhabitants; and to regions currently being used for subsistent mono or polyculture of cassava, banana, cane, and papaya; and, lastly, peridomiciliar refers to areas with the highest anthropisation and housing agglomerations.

**TABLE I:** Schedule for the capture of small mammals in the rural settlement of Rio Pardo (RSRP), municipality of Presidente Figueiredo, State of Amazonas

Expedition	Months	Year	Duration
1st	August-September	2016	23 days
2nd	November	2016	30 days
3rd	May	2017	15 days
4th	September	2017	18 days

Sherman and Tomahawk traps (Live Traps) were set to capture mammals along 16 trails (four in each landscape); the trails were approximately 500 m in length, and each had 13 trap stations spaced at 30 m from each other. Each station had one Sherman (8 × 8 × 23 cm) and one Tomahawk trap (14 × 14 × 40 cm), which were alternatively positioned (either on the ground or suspended at the height of 1.5 m) at each station. The Pitfall traps were also installed using buckets of 100 L measuring 68 cm × 57 cm (height × diameter) each. Three buckets were positioned in-line, equidistant to each other, perpendicularly to the trail pairs in each landscape. Four hundred and sixteen traps were installed in total, 208 Tomahawk and 208 Sherman (104 over each type of landscape) and 24 Pitfalls.

Pieces of pacovan banana were used as bait for the live traps, along with toasted peanut butter and cotton wads moistened in codfish liver oil, which were renewed every day. The traps were reviewed in the mornings to reduce bait loss due to ants. Traps were replenished with new baits if necessary. Pitfalls were also reviewed and dried every day in the event of rainfall. Styrofoam pieces were poured inside the pitfall to allow the animals to emerge in the case of rain. The captured animals were individually transported to the laboratory unit within cloth bags with sizes varying according to the size of the animal. Each bag was labeled with trap type and location.

During the afternoons, back at the laboratory unit, each captured animal was weighed to calculate the doses of anesthesia. After sedation, their tails and ears were inspected for depigmentation, alopecia, and small lesions over the skin. They were labeled thereafter by sex, weight, and size, and then initially identified by genus based on their morphological traits, with the help of taxonomic guides. A cardiac puncture was made to collect material for another study while the animals were under anesthesia, and after euthanasia, the liver of the animals were removed; the livers were added to Eppendorf microtubes with absolute alcohol, and stored at 4ºC.

For anesthetic induction, the animals were individually placed in hermetically sealed plastic boxes to inhale isoflurane. Cotton balls were moistened with the anesthetic and placed in the box such that they were out of the animal’s reach. The animals were then anesthetised with ketamine (200 mg/kg) and xylazine (10 mg/kg) for invasive procedures. Euthanasia was performed with twice the dose of ketamine and xylazine.

In the animal genetics laboratories of National Institute of Research within the Amazon (INPA), the animals were confirmed to the species level through cranial analysis, and added to INPA’s mammal collection.

*Dna extraction and polymerase chain reaction (PCR)* - The DNA of tissue samples was extracted using the DNeasy Blood & Tissue Kit (Qiagen, Hilden, Germany). The tissue was lysed with K proteinase, and the DNA was linked to a membrane in a column, where it was purified and then diluted in a buffer solution. A PCR using the GoTaq Flexi DNA Kit (Promega^®^) with LSPP primer was performed to detect the trypanosomatids. A PCR with the Platinum Kit (Invitrogen^®^) and the kLEISH primer, following the manufacturer’s recommendations, was also performed. Conditions of PCR and the primers used are shown in Table II.^12^ ^,^ ^13^ The DNA with the positive control was extracted from cultures donated by the Tropical Medicine Foundation (FMT). Amplified products were visualised on a 2% agarose gel stained with GelRed.

**TABLE II:** Profile of polymerase chain reaction (PCR) and primers applied for the identification of trypanosomatids in liver tissues of the small rodents collected in the rural settlement of Rio Pardo (RSRP), municipality of Presidente Figueiredo, State of Amazonas

Amplified region	Sequence	PCR cycles	Amplicons
Minicircle of kDNA - Gene miniexon of tripanosomatids (Fernandes et al.^12^ - LSPP)	F5′GGG AAT TCA ATA TAG TAC AGA AAC TG3′ R5′GGG AAG CTT CTG TAC TTT ATT GGT A3′	94°C 5 m/5x 94°C 30 s, 45°C 2 m, 65°C 30 s/25x 94°C 30 s, 50°C 1 m, 72°C 3 m/72°C 10 m, volum final 25 uL	*L. (V.) lainsoni*: 164 pb, *L. (V.) braziliensis* ou *L. (V.) guyanensis*: 253-264 pb, *L. (L.) amazonensis* ou *L. (V.) mexicana*: 315-332 pb e *L. (V.) major* ou *L. (V.) tropica* 435-460 pb
Minicircle of kDNA - Minicircles of *Leishmania* - (Tonelli et al.^13^ - kLEISH)	A5’(C/G)(C/G)(G/C) CC(C/A) CTA T(T/A)T TAC ACC AAC CCC3’ B5’GGG GAG GGG CGT TCT GCG AA3’	94°C 4 m/35x 94°C 30 s, 60°C 30 s, 72°C 30 s/72°C 10 m, volume final 25 uL	120 pb

*Data analysis* - The Rényi test was used to evaluate the diversity and abundance of species of rodents and marsupials across the four landscapes. Permutational multivariate analysis of variance (PERMANOVA) was performed to verify significant differences between landscapes. Kernel maps were created to identify the landscape with a higher IR (within 1 km diameter). Two domiciliary landscape samples were excluded due to the lack of coordinates. All figures were prepared in R Studio v3.5, and the maps were prepared using QGIS v2.18.

*Ethics* - The project was approved by the local SISBIO Nº 54970-1 (general license for animal collection) and by UFOPA’s CEUA Nº 0120180002 (Federal University of West Pará, ethics committee for animal use).

## RESULTS

A total of 135 small wild mammals were captured from 14 genera, 81 (60%) and 54 (40%) of which were from the orders Rodentia and Didelphimorphia, respectively (Table III). Table II presents the abundance of rodents and marsupials within each landscape of the RSRP.

**TABLE III:** Mammals of the orders Rodentia and Didelphimorphia captured by Tomahawk and Sherman traps across four landscapes types in the rural settlement of Rio Pardo (RSRP), municipality of Presidente Figueiredo, Amazonas State

Species	Domicile	Planting	F.+plant.	Forest	N	I	RA	IR
Rodentia								
*Hylaeamys* sp.	1	0	3	2	6	6	0,044	100,00%
*Hylaeamys megacephalus*	1	2	4	1	8	8	0.059	100,00%
*Isothrix pagurus*	0	0	1	0	1	0	0,007	0,00%
*Neacomys paracou*	0	0	30	7	37	30	0,274	81,08%
*Oecomys bicolor*	0	0	0	1	1	1	0,007	100,00%
*Oecomys paricola*	0	0	1	0	1	0	0,007	0,00%
*Oecomys* sp.	0	0	4	0	4	0	0,029	0,00%
*Proechimys cuvieri*	0	0	2	0	2	0	0,014	0,00%
*Proechimys guyannensis*	0	0	1	0	1	1	0,007	100,00%
*Proechimys* sp.	0	2	5	0	7	2	0,051	28,57%
*Rattus rattus*	12	0	0	0	12	11	0,088	91,67%
*Rhipidomys* sp.	0	0	1	0	1	1	0,007	100,00%
SUBTOTAL	14	4	52	11	81	60		74,07%
Didelphimorphia								
*Caluromys philander*	0	0	1	0	1	0	0,007	0,00%
*Didelphis marsupialis*	3	2	6	1	12	2	0,088	16,67%
*Marmosops parvidens*	0	0	1	2	3	1	0,022	33,33%
*Marmosops pinheiroi*	0	0	2	1	3	0	0,022	0,00%
*Marmosops* sp.	0	0	2	3	5	1	0,037	20,00%
*Metachirus nudicaudatus*	0	0	2	2	4	3	0,029	75,00%
*Marmosa demerarae*	1	1	6	4	12	8	0,088	66,67%
*Monodelphis kunsi*	0	0	1	0	1	0	0,007	0,00%
*Monodelphis brevicaudata*	0	1	3	2	6	5	0.044	83,33%
*Philander opossum*	0	1	6	0	7	6	0.051	85,71%
SUBTOTAL	4	5	30	15	54	26		48,15%
TOTAL	18	9	82	26	135	86		63,70%

F.+Plant: forest with planting; N: number of animals; I: infected; RA: relative abundance; IR: infection rate.

The greatest abundance and richness of small animals were captured through pitfall traps, followed by Tomahawks, and Shermans. However, Didelphimorphia had equivalent abundance from Tomahawk and Pitfall traps (Table IV).

**TABLE IV:** Mammals of the orders Rodentia and Didelphimorphia captured by Tomahawk and Sherman traps in the rural settlement of Rio Pardo (RSRP), municipality of Presidente Figueiredo, Amazonas State

Species	P	T	S	Species	P	T	S
*Rodentia*				*Didelphimorphia*			
*Hylaeamys* sp.		1	5	*Caluromys philander*		1	
*Hylaeamys megacephalus*	3	2	3	*Didelphis marsupialis*		12	
*Isothrix pagurus*		1		*Marmosops parvidens*	3		
*Neacomys paracou*	37			*Marmosops pinheiroi*	3		
*Oecomys bicolor*	1			*Marmosops* sp.	5		
*Oecomys paricola*	1			*Metachirus nudicaudatus*	3	1	
*Oecomys* sp.	2		2	*Marmosa demerarae*	2	6	4
*Proechimys cuvieri*	1	1		*Monodelphis kunsi*	1		
*Proechimys guyannensis*	1			*Monodelphis brevicaudata*	5		1
*Proechimys* sp.	1	4	2	*Philander opossum*	2	5	
Rattus rattus		7	5	-			
*Rhipidomys* sp.			1	*-*			
SUBTOTAL	47	16	18	SUBTOTAL	24	25	5
%	58%	19,8%	22,2%	%	44,4%	46,3%	9,3%

P: Pitfall; T: Tomahawk; S: Sherman.

The most abundant species of rodent was *Neacomys paracou*, with 37 samples (45.67%), followed by *R. rattus* with 12 samples (14.81%) and *Hylaemys megacephalus* with eight samples (9.87%). The first was captured in forest landscapes (continuous forest and forest with planting), the second was captured only in the peridomiciliar landscape, and the third was found across all landscapes. *H. megacephalus*, therefore, is supposed to have enhanced movements between the landscape. The richness of rodents was lowest in the planting landscape (1) and highest in the forest with planting landscape (6).

The most abundant marsupial species were *D. marsupialis* and *Marmosa demerarae*, with 12 samples each (22.22%), followed by *Philander opossum* with seven (12.96%), and *Monodelphis brevicaudata* with six samples (11.11%). The first two were captured across all four landscapes indicating enhanced movements across the landscape, the third was captured in the planting and forest with planting landscapes, whereas the last one was found everywhere except in the peridomiciliar landscape. The peridomiciliar landscape had a lowest species richness of marsupials (2) while the forest next to planting had a highest species richness (9).

**Fig. 2:**
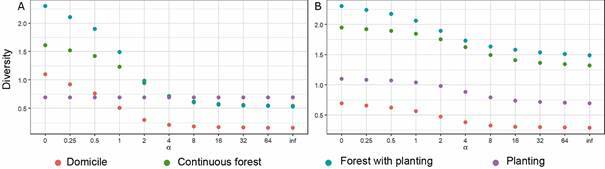
(A) diversity of species from order Rodentia in four distinct landscapes of the rural settlement of Rio Pardo (RSRP), obtained through Rényi tests. Shannon-Weaver index at 1, Simpson index at 2. (B) Diversity for the species of the Didelphimorphia order in four distinct landscapes of the RSRP, Shannon-Weaver index at 1, Simpson index at 2.

The Rényi diversity for Rodentia and Didelphimorphia yielded similar results to the Shannon-Weaver (1) and Simpson (2) indices, indicating that abundance and richness were higher in the forest next to planting landscape, and lower in the peridomiciliar landscape. However, for Rodentia, the Simpson index also showed similar diversity levels in forest next to planting, planting, and continuous forest landscapes (Fig. 2).

Diversity was not significantly different for Didelphimorphia (PERMANOVA, p = 0.12) but was significantly so for Rodentia (PERMANOVA, p = 0.0005) between the four landscapes.

The 14 captured rodent and marsupial genera showed no signs of lesions, all of which were therefore asymptomatic for leishmaniasis.

**Fig. 3:**
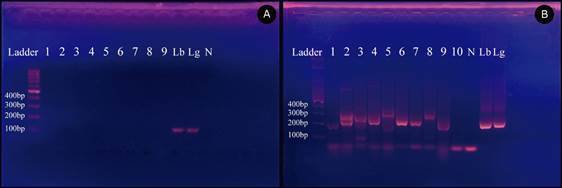
agarose gel electrophoresis 2%. (A) Standard polymerase chain reaction (PCR) amplification of the mini-exon gene available within the minicircle of the kinetoplast using kLEISH primer. (B) Standard PCR amplification of the mini-exon gene available within the minicircle of the kinetoplast using LSPP primer. Both figures: 1,2,3,4,5,6,7,8,9,10: samples, Lb: *Leishmania brasiliensis*, Lg: *L. guyanensis* and N as negative control.

The kLEISH primer showed no DNA amplification in the used samples (Fig. 3A), while the LSPP primer amplified 86 of the samples (63.7%) in varying band sizes. All attained results were compatible with *Leishmania*:^12^ 48 samples (200-265 bp) compatible with *L. braziliensis* or *L. guyanensis*, three samples (160-190 bp) compatible with *L. lainsoni*, 25 samples (300-330 bp) compatible with *L. amazonensis* or *L. mexicana*, and one sample (430-470 bp) compatible with *L. major* or *L. tropica.* Nine other samples showed amplicons close to 800 and 200-265, or 300-330 bp (Fig. 3B).

The IRs for *Leishmania* were higher in the peridomiciliar landscape (92.9%), followed by continuous forest (81.8%), forest planting (69.2%), and planting (50%) landscapes. The IR for marsupials was higher in continuous forest and forest with planting, both with 53.3%, followed by domicile (25%), and planting (20%).

**Fig. 4:**
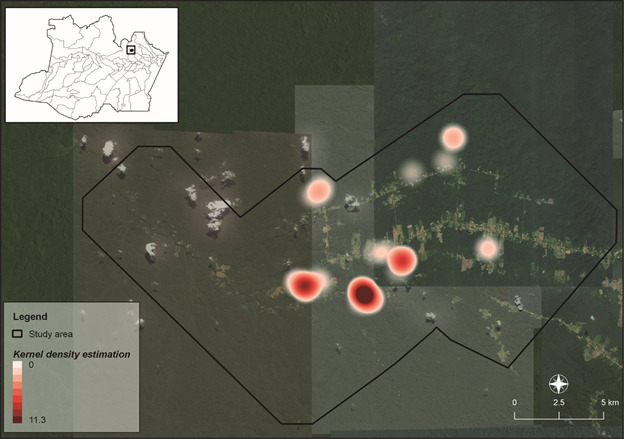
Kernel analysis shows the distribution of *Leishmania* infected rodents in different landscape within the rural settlement of Rio Pardo (RSRP), municipality of Presidente Figueiredo, Amazonas State.

*Rattus rattus* was found only within the peridomiciliar landscape, with an IR of 91.67% (11/12). The genus *Hylaeamys* was captured in all four landscapes with 100% IR (14/14). *Neacomys* sp. captured in forest and forest with planting landscapes showed an IR of 81.08% (30/37). The distribution of infected rodent species is shown in Fig. 4.

**Fig. 5:**
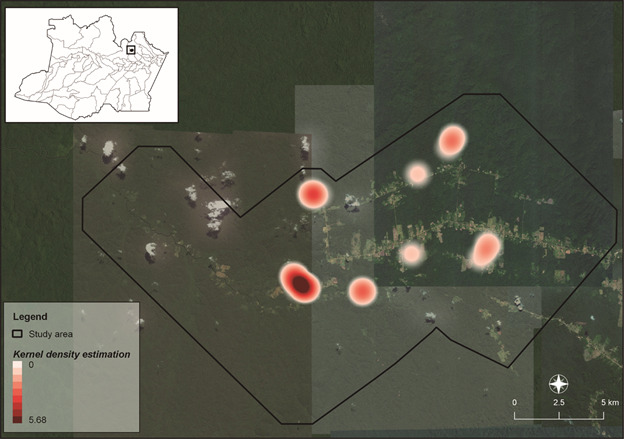
Kernel analysis shows the distribution of *Leishmania* infected marsupials in different landscape of the RSRP, municipality of Presidente Figueiredo, Amazonas State.

Among marsupials, the species *Philander opossum* presented a higher IR (85.71%), most of which were captured in the forest with planting landscape. *M. brevicaudata* had an IR of 83.33% (5/6), and *M. demerarae* of 66.67% (8/12); both were collected from all landscapes except planting. *Metachirus nudicaudatus* presented an IR of 75% (3/4) and was captured in the forest landscape. *D. marsupialis* was present in all four landscapes with a lower IR of 16.67% (2/12). The distribution of infected marsupials is shown in Fig. 5.

Although the Kernel analysis featured a high density of infected rodents and marsupials in forests with planting (Figs 4, 5), there was a low correlation between the total *Leishmania*-infected Didelphimorphia and the different landscapes (rho = 0.344; p = 0.09212); Rodentia had the lowest correlation (rho = 0.267; p = 0.04224). The infected species were distributed in all the studied landscapes with the highest number within the forest with planting landscape.

## DISCUSSION

This is the first study on the ecology of the landscapes within the State of Amazonas, involving a systematic capture of 135 small terrestrial mammals of the orders Rodentia and Didelphimorphia. A total of seven genera and eight species from the order Rodentia were captured as well as seven genera and nine species from the order Didelphimorphia. Only one genus from each order showed no infection (*Isothrix pagurus* and *Caluromys philander*).

The pitfall traps used in this study were more efficient in both abundance and richness. The results for Didelphipmorphia were the same for the Pitfall and Tomahawk traps. Some species were captured exclusively through one trap type, which was the case for *N. paracou* and *D. marsupialis* in Pitfall and Tomahawk traps, respectively. The evaluation of trap efficiency is not one of the objectives of this study. However, in accordance with other studies, it is still recommended that all three trap-types be used together for comprehensive sampling of small mammals.^14^

The results showed that the majority of abundance and richness of small mammals occurred in the forest with planting landscape, while the minority occurred in the peridomiciliar landscape. Our results are similar to those of Pardini et al.,^15^ who found diversity loss in fragmented landscapes. We hypothesise that rodents and marsupials are being lured to forests near plantings due to their broader availability of food. However, more studies are necessary to understand this higher diversity in forests near plantings.

The anthropisation process reduces diversity but also promotes selection of generalist species, creating the phenomenon known as “dilution effect”, which generally increases emerging and re-emerging diseases when selected species are competent.^16^ In addition, the highest IRs for this study occurred in the peridomiciliar landscape (92.9%, Rodentia order), where only two genera were captured: *R. rattus* and *Hylaemys* sp., with IRs of 91.67% and 100%, respectively. Even with the same anthropic effects as with Rodentia, the Didelphimorphia presented higher IRs in the forest landscape. There was no correlation between the IR and the landscape, indicating that the infection risk for *Leishmania* was similar for all studied landscapes.

PCR results using the LSPP primer generated amplicons that suggested the activity of different *Leishmania* species within the rodents of the region. Fernandes et al.^12^ used LSPP primers to identify *Leishmania*, while Murthy et al.^17^ differentiated *T. cruzi* and *T. rangeli* with them. LSPP is a primer that amplifies the mini-exon gene that is present within the minicircle of the kinetoplast.^12^ Highly preserved regions are used to make this differentiation, 39 preserved nucleotides^12^ in the case of *Leishmania* sp. and 22 preserved nucleotides in the case of *Trypanosoma* sp.^17^ Between these highly conserved regions lies a spacer of variable size that allows differentiation of species.^12^ ^,^ ^17^ Both authors used cloning techniques to identify species.

This study did not cover cloning or sequencing, therefore, the identification of the infection at the species level was not attained. Cloning is an expensive and time-consuming technique generally used for *Leishmania* typification. Although, amplification pattern comparison with known standards have been widely used and with a good correlation when clonning and sequencing was applied. Moreover, there were difficulties in obtaining, from the field samples, amplifications of targets with few (or single) copies and no (or few) variance between genes, indicating the need to make a culture of the cells.

Molecular probes based on the mini circles (the largest component of the kDNA with approximately 10.000) have high sensitivity to the use of initiators based on sequences that have more than one copy per cell. These sequences include the kinetoplast and families of multi-copied genes that leverage studies related to the detection and counting of parasites.^18^ Because no sequencing was performed, it is safe to consider the opportunity to identify the species of *Leishmania*, given the high sensitivity of this primer to the Trypanosomatids and the size of amplicons available for *Leishmania*.

The inaccurate specificity of the LSPP primer can explain the compatibility of one sample (430-470 bp) with *L. major or L. tropica*, which does not occur in Brazil; the same is applicable for nine amplicons migrating at approximately 800 bp. In contrast, the negative results using the kLEISH primers could contest the results using LSPP primers, which can only be verified by sequencing. Moreover, denying the LSPP results would mean disregarding its sensitivity and the amplifications at *Leishmania* equivalent levels; most importantly, disregarding the LSPP results would mean disregarding the fact that they were indicating other members of the Trypanosomatidae family.

The results of this study highlight the susceptibility of rodents and marsupial to *Leishmania* infection, as 12 out of 14 small mammal genera were infected.^6^ ^,^ ^7^ ^,^ ^10^ ^,^ ^19^ ^,^ ^20^ According to McFarlane,^21^ synanthropic animals are more prone to being hosts of zoonotic diseases than wild ones, which supports our findings: *R. rattus* was the most frequent species in the peridomiciliar landscape (100%) with an IR of 91.76%. This species needs to be investigated further. In this study, we could not declare *R. rattus* as a reservoir because PCR was performed on liver samples. However, the high IR of this species, together with its area inside or near domiciles, can justify the need for such investigation owing to the risks it poses to humans. *R. rattus* specimens have been found with natural infections of *L. braziliensis* in the State of Ceará, and *L. amazonensis* in the State of Paraná, in urban and suburban regions.^7^ ^,^ ^22^

Another important finding is that *Hylaemys* sp. and *H. megacephalus* were found with IRs of 100%. The latter is widely spread across Brazil, and is well adapted to modified/degraded landscapes (IUCN).^23^ In the present study, a similar result was observed, as this species was collected in all four landscapes studied in the RSRP. The *Hylaemys* species,- previously acknowledged as *Oryzomys* or, in Brazil, as *Oryzomys capito*, *O. concolor*,^24^ *O. nigripes*,^25^ and *O. subflavus*,^26^ - were all found to be infected with *L. braziliensis*. *Oryzomys acritus* and *O. nigripes* were found to be infected with *L. braziliensis* in Bolivia, and *Oryzomys melatonis* and *O. capito* were found to be infected by *L. mexicana* in Mexico and Panamá, respectively.^27^ ^,^ ^28^

*Neacomys* sp. was the most abundant rodent species and was captured exclusively in forest landscapes (continuous forests and forest with plantings) with IRs of 81.08%. Given its localisation in the RSRP, its role in the transmission cycle for the disease seems to be restricted to wild environments.

Marsupials presented a lower IR (48.1%) than rodents, with the highest IR in the forest landscape. *D. marsupialis* was found in all the studied landscapes, confirming its high adaptability to different ecological areas.^6^ Only two samples of *D. marsupialis* were infected in this study, with an IR of 16.67%, similar to previously reported (20%) in the Reserva Ducke.^8^

In a study conducted in an urban forest fragment in Salvador, Bahia, 22 *D. marsupialis* samples were collected, eight of which were infected by *T. cruz*i and 10 with *Leishmania* sp.^10^ *D. marsupialis* is considered as a natural reservoir for *T. cruzi* and has been found in many places in Brazil^29^ ^,^ ^30^ ^,^ ^31^ and in Venezuela.^32^

Our findings indicate a broader diversity of rodents and marsupials in forests with planting and a lesser one in peridomiciliar landscapes. However, the high IR in all landscapes show that there is little difference in the risk from circulating trypanosomatids between the four landscapes. The identification of *Leishmania* species must be accurate to establish the real risk offered by rodents for the synanthropisation of ATL in the Amazon region.
